# A Very Unusual Presentation of Metastatic Colon Cancer

**DOI:** 10.5402/2011/531803

**Published:** 2011-06-23

**Authors:** Farzan Fahrtash, David Chan, Andrew Colebatch, Joseph Rutovitz

**Affiliations:** ^1^Department of Medical Oncology, Westmead Hospital, Sydney, NSW 2145, Australia; ^2^Department of General Medicine, Hornsby Hospital, Palmerston Road, Hornsby, Sydney, NSW 2077, Australia; ^3^Department of Anatomical Pathology, Royal North Shore Hospital, Sydney, NSW 2065, Australia

## Abstract

This case highlights two very rare complications of metastatic colorectal carcinoma. It describes a 59 year old female with both cutaneous and endometrial metastases from colorectal carcinoma. While both of these presentations are very unusual, they highlight the need to be vigilant about the detection of metastatic complications during follow up.

## 1. Introduction

**Figure 1:**
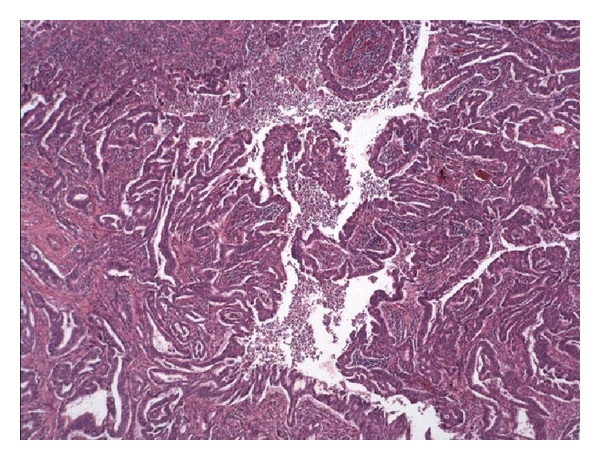
Original rectosigmoid adenocarcinoma.

A previously well 59-year-old lady presented to her gastroenterologist with per rectal bleeding. Apart from intermittent inhaled salbutamol for asthma, she takes no medications and has no allergies. She is a lifelong nonsmoker and consumes 30 g of alcohol per week. She has no family history of note. Colonoscopy demonstrated a tumour at the rectosigmoid junction (see Figure 1). 

A staging CT of Chest/Abdomen/Pelvis revealed no obvious distal metastatic disease. Anterior resection and cholecystectomy was performed in June 2006 without complication. Histopathology demonstrated moderately well differentiated rectosigmoid adenocarcinoma. Resection margins were free of tumour. Both lymphatic and venous invasion were noted; 17 of 18 regional lymph nodes including the apical gland contained metastatic tumour. She was staged as T3N2M0 (IIIb). She received adjuvant FOLFOX-6 (Fluorouracil, Oxaliplatin, bolus Fluorouracil, and Leucovorin) for six months. Full course and doses were achieved with only mild thrombocytopaenia.

Repeat CT scan at end of chemotherapy (January 2007) demonstrated two 5 mm lesions in the left lower lobe and right middle lobe of the lung. While metastatic disease could not be ruled out, the lesions were thought to be granulomatous in nature. Two low attenuation areas were identified in the liver posteromedially, measuring 8 mm and 15 mm in diameter. A para-aortic lymph node measured 6 mm.

Progress CT in July 2007 demonstrated increase in the para-aortic lymphadenopathy. Repeated 4 months later, a new lobulated 5 mm nodule was noted in periphery of right upper lobe along with more extensive para-aortic and left common iliac lymphadenopathy, suspicious of recurrent disease. PET scanning demonstrated FDG avid disease involving lymph node groups both above and below the diaphragm, and in particular the left para-aortic chain. CT confirmed extensive left para-aortic lymphadenopathy with more focal nodal masses posterior to left renal vein and at common iliac bifurcation. 

A needle biopsy of the para-aortic lymph node confirmed adenocarcinoma consistent with prior colonic primary.

She received a further 6-month course of FOLFOX-6 from 3/3/2008 to 6/8/2008; again achieving a good partial response with resolution of the adenopathy and stable disease in the other measurable lesions.

In November 2008 the patient reported left leg lymphoedema. Progress imaging and Doppler Ultrasound of legs revealed no new pathology. The oedema progressed and spread to the thigh, with left iliac fossa tenderness felt at the same time. CEA had increased to 8.8.

The patient developed per vaginal bleeding in January 2010. Pelvic ultrasound demonstrated a thin and regular endometrium. CT showed no evidence of recurrence at surgical site, as well as decreased size of both right middle lobe lung lesion and left external iliac lymph nodes. She was referred for uterine curettage, performed in February 2010. This demonstrated scanty disintegrating adenocarcinoma cells infiltrating the lower uterine segment. The malignant cells were positive for CK20 and negative for CK7, supporting diagnosis of metastatic colonic carcinoma. P16 was negative and CEA/mucin stains were difficult to interpret due to degenerative changes. 

She was referred for gynaecological assessment in February 2010. Per vaginal examination showed the uterus to have a firm and nodular consistency. 

Along with the PV bleeding, the patient reported right upper back pain in a radicular pattern from the lower cervical spine to the right proximal humerus. There was no neurological deficit. CT on same day showed a destructive lesion in C7 vertebral body on right side with pathological fracturing. Whole body bone scan with SPECT 19/2/10 showed intense abnormality in body C7 consistent with a metastatic lesion. MRI of the spine 19/2/10 showed pathological superior endplate fracture associated with metastatic deposit in the C7 vertebral body. Some bone retropulsion into canal was present without significant canal stenosis/cord impingement. Marked right-sided C8/T1 foraminal stenosis was noted with possible right C7 radicular impingement.

The patient commenced palliative radiotherapy to the cervical spine in 10 fractions of 30 Gy. She tolerated this fairly well with minor dermatological reactions. 

**Figure 2:**
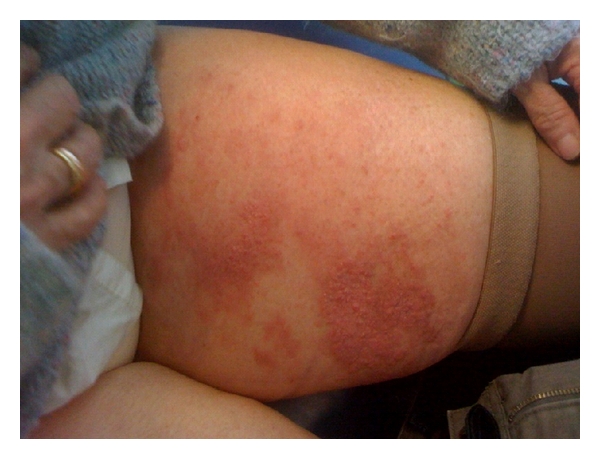
Skin lesion.

**Figure 3:**
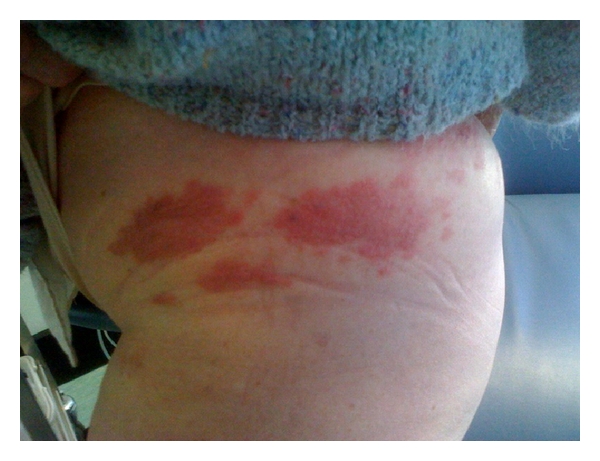
Skin lesion.

**Figure 4:**
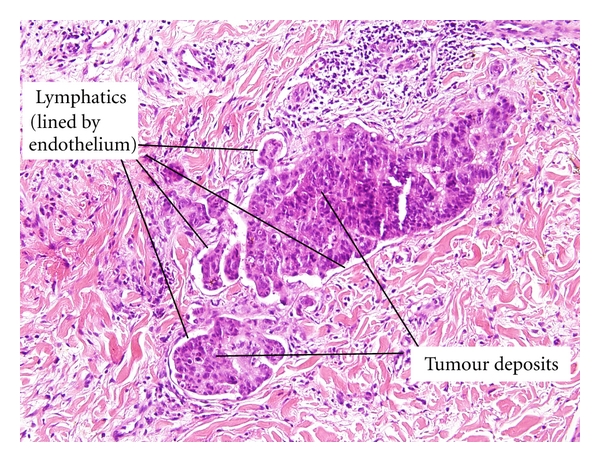
Histopathology of skin biopsy showing multiple micronodules of pleomorphic cells with some tumour nodules within lymphatic channels consistent with metastatic spread of colorectal carcinoma.

A dermatitic like eruption on left upper/inner thigh and buttock was noted in April 2010, which did not respond to treatment with topical steroids (see Figures 2 and 3). She was reviewed by a dermatologist and punch biopsy of left upper thigh was carried out on 28/4/10. This showed multiple micronodules pleomorphic cells in superficial and mid dermis, showing focal ductular differentiation. The biopsy was in keeping with metastatic adenocarcinoma (see Figure 4).

She had 3 cycles of FOLFOX, however, this was poorly tolerated due to anaphylactic reactions. She has since commenced a combination of fluorouracil, folinic acid, and irinotecan.

## 2. Discussion

Colorectal cancer is the second most prevalent malignancy in Australia (excluding nonmelanocytic skin cancers) [1]. Approximately 20 percent of patients have distant metastatic disease at the time of presentation [2]. Carcinoma can spread by lymphatic, haematogenous, contiguous and transperitoneal routes. The most common metastatic sites are the regional lymph nodes, liver, lungs, and peritoneum. Consequently, patients may present with signs or symptoms referable to these areas. There are recognized unusual presentations of CRC. These include local invasion causing malignant fistula formation into adjacent organs, such as bladder or small bowel. Fever of unknown origin, intra-abdominal, retroperitoneal, or abdominal wall abscesses can often be the first manifestation.

CRC proves to be the aetiology in 6% of adenocarcinomas of unknown primary sites [3].

Our patient presented with spinal, endometrial, and cutaneous involvement. These presentations are very rare individually, however, there has been no previous case report of a patient with both endometrial and cutaneous features. 

Brand et al. reported a series of 6 patients presenting with per vaginal bleeding in the setting of recurrent colon cancer [4]. Only one of the patients in that series received adjuvant chemotherapy prior to per vaginal bleeding. Despite aggressive chemo-radiation or surgical management, all patients had recurrence of colonic carcinoma. 

Hu et al. found 124 cutaneous metastases from a series of 12.146 patients with internal malignancies (1%) [5]. 16 of these cases originated from colorectal cancer, equating to a rate of cutaneous metastasis of 0.81%. Other authors have found rates of cutaneous metastasis between 2.3 and 6% [6–8]. Cutaneous metastases most often occur on a site relatively close to the internal primary. Skin metastases from the breast usually occur on the chest, the lung to the chest and upper extremities, and the gastrointestinal tract to the abdomen [9]. These metastases usually manifest as a rapidly growing, mobile nodule [10]. On immunohistochemical staining, CK20 positivity often correlates with colorectal tumours [11]. 

 This case highlights the importance of paying close attention to symptoms and signs in patients with a past history of internal malignancy. It is important to maintain a high index of suspicion to clinical features that may represent a metastatic process. Furthermore, tissue diagnosis to confirm the aetiology of a symptom can prove very useful, as shown in this case.
